# Meta-analysis of Prevalence and Risk Factors for Delirium After Transcatheter Aortic Valve Implantation

**DOI:** 10.1016/j.amjcard.2018.08.037

**Published:** 2018-12-01

**Authors:** Erica Tilley, Peter J. Psaltis, Tobias Loetscher, Daniel H. Davis, Stephanie L. Harrison, Susan Kim, Hannah A.D. Keage

**Affiliations:** a Cognitive Ageing and Impairment Neurosciences Lab, University of South Australia, Adelaide, South Australia, Australia; b School of Medicine, University of Adelaide, Adelaide, South Australia, Australia; c Vascular Research Centre, Heart Health Theme, South Australian Health and Medical Research Institute, SAHMRI, Adelaide, South Australia, Australia; d MRC Unit for Lifelong Health and Ageing at UCL, London, United Kingdom; e Department of Rehabilitation, Aged and Extended Care, Flinders University, Adelaide, South Australia, Australia; f Flinders Centre for Epidemiology and Biostatistics, College of Medicine and Public Health, Flinders University

## Abstract

Delirium is a severe and common complication following transcatheter aortic valve implantation (TAVI). We sought to identify the prevalence and risk factors associated with the development of postprocedural delirium in patients aged over 60 years who underwent elective TAVI for aortic stenosis. Overall, 1,051 articles were searched, from which 9 studies were included. The prevalence of delirium following TAVI was higher in studies that assessed delirium for a minimum of 3 consecutive days (24.9%) compared with the studies that did not (2%). There were large effect sizes (*d* > 0.8*)* for 3 risk factors: acute kidney injury (odds ratio [OR] 5, p < 0.001), transapical approach (OR 4, p < 0.001) and carotid artery disease (OR 4, p < 0.001), whilst small effect sizes were found for a history of atrial fibrillation, prior stroke/transient ischemic attack, peripheral artery disease, hypertension, and prior cognitive impairment. In conclusion, 23% of patients 60 years and over who underwent TAVI experience delirium, a preventative cause of cognitive impairment and dementia. Recognition of risk factors for delirium after TAVI, such as a history of carotid artery disease, development of acute kidney injury, or use of a transapical approach, provides an opportunity to implement proven delirium preventative measures.

Delirium (a deficit of attention with an acute and fluctuating course)^1^ is a common complication following transcatheter aortic valve implantation (TAVI). Patients with delirium following TAVI have twice the length of hospital stay,^2^ nearly 3 times the risk of increased hospital readmissions and mortality within 180 days of the procedure,^3^ and are twice as likely to be admitted to a rehabilitation facility,^2^ compared with their nondelirious counterparts. In the general population aged over 85 years, delirium has been associated with increased risk of incident dementia (odds ratio [OR] 9, 95% confidence interval [CI] 2.1 to 35.1) and cognitive decline (OR 3, 95% CI 1.4 to 5.5).^4^ In older hospitalized adults, there is evidence that delirium is preventable in 20% to 30% of cases.^5^, ^6^ A study using multicomponent interventions (orientation protocol, cognitively stimulating activities, early mobilization, and nonpharmacologic sleep, vision, hearing, and dehydration protocols), found the incidence of delirium was significantly lower in the intervention group than the usual-care group (10% vs 15%, p = 0.02).^6^ It is therefore important to identify those at high-risk of developing postprocedural delirium following TAVI in order to target potentially preventative measures. This systematic review set out to examine the period prevalence and risk factors for delirium in TAVI patients.

## Methods

The primary outcome of interest was the period prevalence of delirium identified using a standardized method such as the Confusion Assessment Method (CAM)^7^ or the Diagnostic and Statistical Manual of Mental Disorders-IV (DSM-IV).^8^ Studies published in English from the time of the first TAVI procedure in 2002 until 12 February 2017 were included. Studies were excluded where there were mixed sample studies (e.g. TAVI and surgical aortic valve replacement) and the results for different operative types were not presented independently. Key search terms and databases searched have been included in the Supplement. Two independent reviewers (ET and SH) screened studies at title/abstract level and at full text level, assessed the quality of the evidence using the Joanna Briggs Institute (JBI) Critical Appraisal Checklist for Studies Reporting Prevalence Data^9^ (Supplement Table 1), and extracted data using a predefined data extraction template. Any disagreements were resolved through discussion.

**Table 1:** Summary of the included studies. CAM = Confusion Assessment Method; DSM-IV = Diagnostic and Statistical Manual of Mental Disorders-IV; P = prospective; PO = postoperatively; R = retrospective

Study	Country	Study type	N	Mean age years(SD)	Delirium(%)	Delirium assessment	Timing of delirium assessment
Abawi (2016)	Netherlands	R	268	80 (7)	36 (13%)	DSM-IV	Hospital stay.
Assmann (2016)	Netherlands	P	89	80 (6)	25 (28%)	DSM-IV	Hospital stay.
Eide	Norway	P	63	85 (3)	28 (44%)	CAM	1 to 5 days PO.
Erdoes (2012)	Switzerland	P	44	78 (6)	0 (0%)	CAM	Prior to, andat 1-, 4-, 5- and 6-days PO.
Fanning (2016)	Australia	P	40	82 (7)	1 (3%)	CAM	Day before,and 3 days,6 weeks and6 months PO.
Huded (2016)	USA	R	294	83 (8)	61 (21%)	CAM, CAM-ICU and DSM-IV	Hospital stay.
Maniar (2016)	USA	R	168	81 (8)	49 (29%)	CAM-ICU	Hospital stay.
Sharma (2016)	Canada	P	210	84 (6)	45 (21%)	CAM and CAM-ICU	1 to 3 days PO.
Tse (2015)	Canada	R	117	81 (8)	32 (27%)	DSM-IV	Hospital stay.

Meta-analyses were carried out using the Comprehensive Meta-Analysis Software (Version 3.3.070, Biostat, Englewood, NJ). Random-effects modeling was used as there was insufficient overlap in study methodology and statistical heterogeneity was high (estimated using chi-squared). Effect sizes were calculated by converting OR to Cohen's *d* and were classified as either large (*d* > 0.8), medium (*d* > 0.5) or small *(d* > 0.2).

## Results

**Figure 1:**
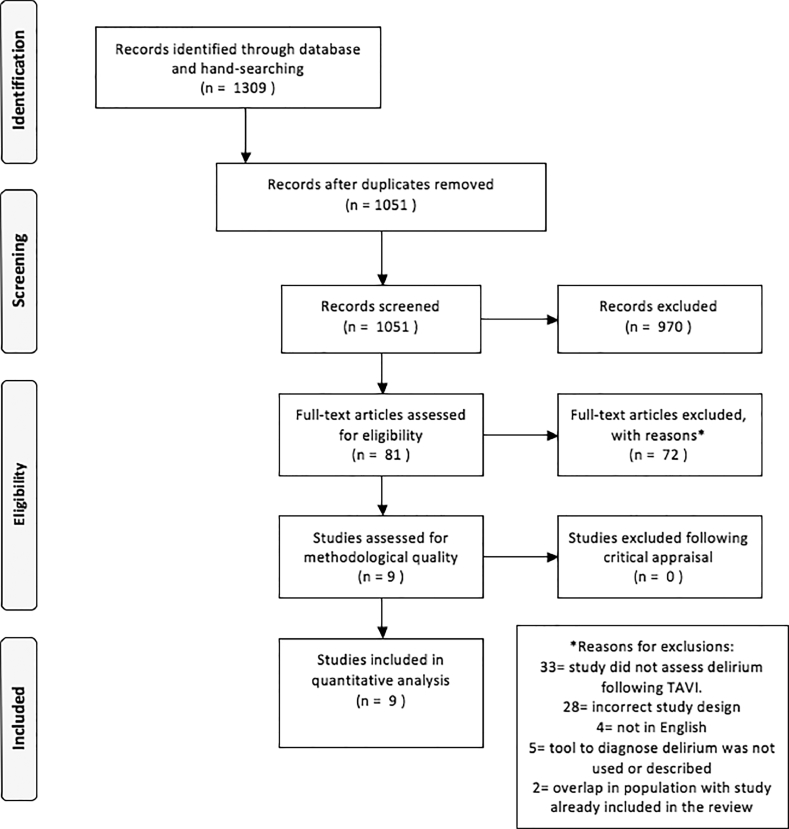
PRISMA search protocol. TAVI = transcatheter aortic valve implantation.

A total of 1,309 titles were screened in this review (Figure 1). Following the removal of duplicate articles, 81 articles were retrieved for full text detailed examination. In total, 9 studies were included in the final review and meta-analyses (see Table 1 for an overview of the included studies). No studies were excluded on the basis of methodological quality (Supplement Table 1). All 9 studies used an appropriate sample frame, described the study subjects and setting in detail and used valid methods for the identification of delirium. The study by Maniar et al. (2016)^10^ scored the highest (7 of 9) on the JBI Critical Appraisal Checklist for Studies Reporting Prevalence Data.^9^ It was the only study to report confidence intervals on the prevalence of delirium. None of the included studies were adequately powered to identify the prevalence of delirium (See Supplement Table 1 for calculations). The largest sample size (n = 294) was in the study by Huded et al.^2^

**Figure 2:**
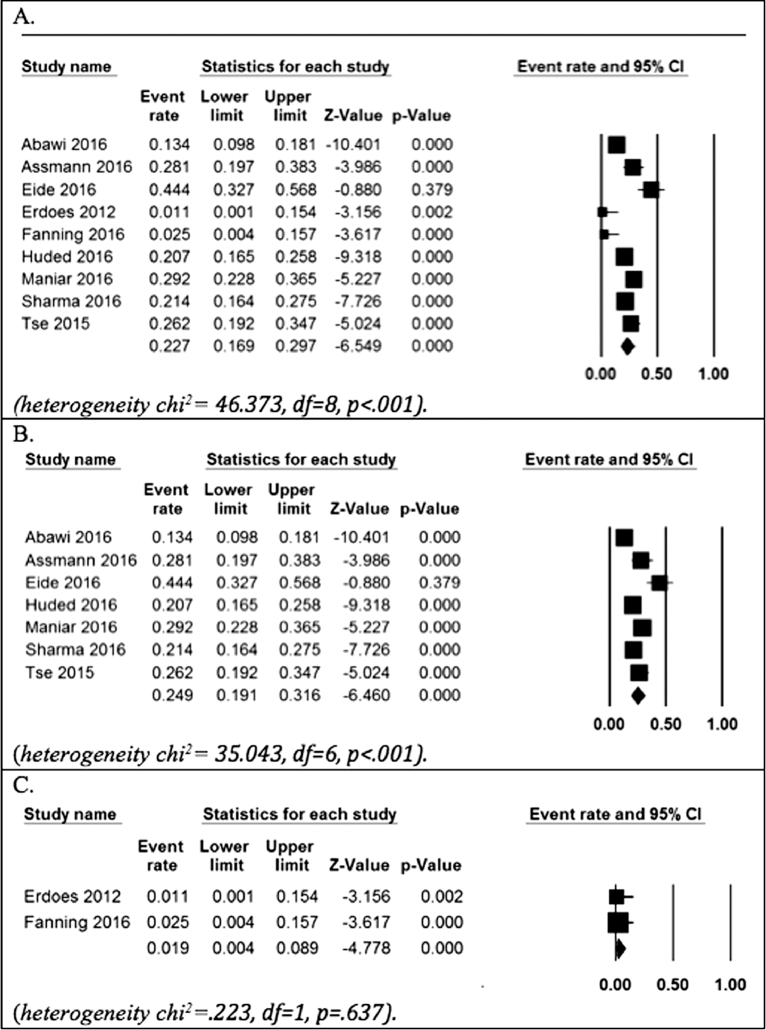
Forest plots; *(A)* Overall pooled prevalence, *(B)* Pooled prevalence for studies where delirium was assessed for 3-days postoperatively and *(C)* Pooled prevalence for studies where delirium was assessed for 3-days postoperatively. df = degrees or freedom.

The results from the meta-analyses on the prevalence of delirium following TAVI are presented in Figure 2. The pooled prevalence of delirium following TAVI was 23% (95% CI 16.9 to 29.7). The pooled prevalence of delirium of studies that assessed for delirium at least once a day for a minimum of 3 days following the TAVI procedure was 24.9% (95% CI 19.1 to 31.6); whereas the pooled prevalence of studies^11^, ^12^ that did not assess for delirium daily for all 3 days following the TAVI procedure was 2% (95% CI 0.4 to 8.9). The prevalence of delirium following TAVI using the CAM^7^ or CAM-ICU^13^ was 23% (95% CI 15.4 to 33.2), and using the DSM-IV^8^ was 21% (95% CI 15.4 to 28.5).

Risk factors for the development of delirium post TAVI were assessed in 7 studies.^2^, ^10^^,^^14^, ^15^, ^16^, ^17^, ^18^ Meta-analyses on pre- and periprocedural variables for the development of delirium are presented in Table 2 and Supplement Table 2. The meta-analyses contain a mix of mainly univariate OR data and multivariate OR data (from the study by Sharma et al.^15^). Pooling of post-TAVI risk factors (i.e. the development of cardiac tamponade, atrial fibrillation, infection, and hospital length of stay) for the development of delirium following TAVI was not possible as only one study reported on each postprocedural variable.

**Table 2:** Meta-analyses on pre- and periprocedural variables for the development of delirium following TAVI

Variable	Studies	N	OR or *SDM (95% CI)	p value	Cohen's d	Heterogeneity Chi**^2^**
Age	Abawi, Eide, Huded and Maniar.	793	0.10* (−0.07-0.27)	0.261	0.04	2.73, df = 3, p = 0.435.
ASA	Eide and Maniar.	231	0.78 (0.31-1.95)	0.595	−0.14	0.00, df = 1,p = 0.951
AF	Abawi, Huded, Maniar and Sharma.	940	1.9*2 (1.37-2.69)	<0.001	0.36	0.39, df = 3, p = 0.943
AVA	Eide and Huded.	357	−0.09* (−0.34-0.15)	0.466	0.04	0.17, df = 1, p = 0.679
BMI	Abawi and Maniar.	436	−0.09* (−0.33-0.15)	0.456	0.04	0.50, df = 1, p = 0.480
Carotid artery disease	Abawi and Sharma.	478	4.34 (2.17-8.68)	<0.001	0.81	0.42, df = 1, p = 0.519
CI	Huded and Tse.	411	2.29 (1.08-4.88)	0.031	0.46	0.63, df = 1, p = 0.429
COPD	Abawi, Assmann, Huded, Maniar and Sharma.	1029	1.08 (0.75-1.56)	0.688	0.04	2.51, df = 4, p = 0.643
CAD	Abawi, Huded, Maniar and Tse.	847	1.39 (0.86-2.24)	0.183	0.18	3.14, df = 3, p = 0.370
Diabetes	Abawi, Assmann, Huded and Maniar.	819	1.02 (0.71-1.46)	0.910	0.01	0.37, df = 3, p = 0.947
EuroSCORE	Abawi and Eide.	331	0.24 (−0.05-0.52)	0.108	0.09	0.79, df = 1, p = 0.373
GFR	Abawi and Maniar.	436	−0.07 (−0.57-0.43)	0.791	0.01	4.28, df = 1, p = 0.039
Hemoglobin	Huded and Maniar.	462	−0.08 (−0.30-0.13)	0.448	0.04	0.81, df = 1, p = 0.369
Hypertension	Abawi, Huded, Maniar and Tse.	847	1.75 (1.08-2.84)	0.024	0.31	2.93, df = 3, p = 0.403
LVEF	Eide and Huded.	357	−0.01 (-0.25-0.24)	0.955	0.00	0.01, df = 1, p = 0.921
Men	Abawi, Assmann, Eide, Huded and Maniar.	882	1.15 (0.83-1.59)	0.398	0.08	3.01, df = 4, p = 0.555
NYHA III-IV	Abawi, Assmann, Eide and Maniar.	588	1.15 (0.70-1.89)	0.574	0.08	2.27, df = 3, p = 0.518
Peripheral artery disease	Abawi, Huded, Maniar, Sharma and Tse.	1057	1.87 (1.02-3.41)	0.043	0.34	12.22, df = 4, p = 0.016
Prior CABG	Abawi and Huded.	562	0.84 (0.51-1.38)	0.481	−0.01	0.02, df = 1, p = 0.901
Stroke/TIA	Abawi, Huded, Sharma and Tse.	889	1.94 (1.25-3.04)	0.004	0.37	1.70, df = 3, p = 0.636
Acute Kidney Injury	Huded and Maniar.	462	4.67 (2.24-9.74)	<0.001	0.85	0.01, df = 1, p = 0.910
Transapical approach	Abawi, Huded, Maniar and Tse.	1057	4.49 (2.2`5-8.98)	<0.001	0.83	16.00, df = 4, p = 0.003

ASA = American Society of Anesthesiologists Physical Status Class 4; AF = atrial fibrillation; AVA = aortic valve area; BMI = body mass index; CABG = coronary artery bypass grafting; CAD = coronary artery disease; CI = cognitive impairment; COPD = chronic obstructive pulmonary disease; EuroSCORE = European system for cardiac operative risk evaluation; GFR = glomerular filtration rate; LVEF = left ventricular ejection fraction; NYHA III-IV = New York Heart Association Class of Heart Failure III-IV; OR = odds ration; SDM = standard difference of means, and TIA = transient ischemic attack.

Independent analyses identified that the prevalence of delirium was significantly associated with 6 preprocedural risk factors. The presence (vs the absence) of carotid artery disease (OR 4, 95% CI 2.2 to 8.7, p <0.001) had the largest impact on delirium (*d* = 0.81); while the presence of atrial fibrillation, cognitive impairment, hypertension, peripheral artery disease, and prior stroke/TIA displayed a smaller association with delirium (*d* < 0.2).

Increased age, body mass index, European system for cardiac operative risk evaluation, left ventricular ejection fraction, aortic valve area, glomerular filtration rate, male sex, chronic obstructive pulmonary disease, coronary artery disease, prior coronary artery bypass grafting, diabetes, hemoglobin, New York Heart Association Class of Heart Failure III-IV or American Society of Anesthesiologists Physical Status Class 4 were not significantly associated with increased risk of developing delirium following TAVI.

There were 2 periprocedural risk factors that were significantly associated with delirium following TAVI: acute kidney injury (OR 5, 95% CI 2.2 to 9.7, p < 0.001) and transapical approach (OR 4, 95% CI 2.3 to 9.0, p < 0.001). Both risk factors had a large impact on the presence of delirium (*d =* 0.85 and *d =* 0.83 respectively).

## Discussion

This systematic review found that nearly 1-in-4 older adults developed delirium post TAVI. A range of risk factors for the development of delirium post TAVI were identified; acute kidney injury (5 times the risk), transapical approach (4 times the risk), a history of carotid artery disease (4 times the risk), and 2 times the risk for the following factors: prior cognitive impairment, atrial fibrillation, prior stroke/TIA, peripheral artery disease, and hypertension. Taken together, these findings have highlighted that delirium affects a large proportion of older adults, and that a range of risk factors may be important for clinical planning and management of this severe medical complication.

The time-frame for the assessment of delirium post TAVI was found to be important. Two studies identified that delirium prevalence peaks at 2 days post TAVI.^16^^,^^17^ The prevalence of delirium following TAVI was higher in studies that assessed delirium for a minimum of 3 consecutive days (25%) compared with the studies that did not (2%).^11^, ^12^

The prevalence of delirium in TAVI patients is higher in reported figures in otolaryngological (12%) and general surgery cohorts (13%), and less than in cardiac surgeries (up to 51%).^19^ This may be due to inherently different patient profiles at baseline and differences associated with a minimally invasive procedure compared with cardiac surgery requiring sternotomy. Increased age and multiple comorbidities are known risk factors for the development of delirium across a range of hospital settings.^5^ TAVI patients are typically older and have multiple comorbidities compared with patients who underwent surgical aortic valve replacement.^20^ In addition, risk factors for aortic stenosis^21^, ^22^ overlap with risk factors for dementia (including hypertension,^23^ diabetes,^23^ and obesity^23^), which in turn are associated with increased risk for delirium.^24^ How the rates of delirium following TAVI, a minimally invasive procedure, compared with surgical aortic valve replacement is beyond the scope of this review, but one study suggests the prevalence of delirium following surgical aortic valve replacement may be as high as 66%.^14^

The identification of risk factors is critical, as it enables for known delirium intervention strategies^6^ to be implemented in those who will benefit most. The transapical approach (a more invasive procedure where the aortic valve is accessed directly through the left ventricular apex rather than the femoral artery)^25^ carried 4 times the delirium risk as compared with the transfemoral approach in our study. The underlying mechanism behind the difference in delirium risk is unclear, largely because no studies examining delirium as an outcome have randomized participants between the two types of access. There may be a tendency for patients believed to be at increased risk of cerebral emboli to be selected for the transapical approach rather than transfemoral approach despite inconclusive evidence that the transapical approach reduces the rate of new cerebral emboli.^26^ Patients selected for the transapical approach also tend to have higher rates of peripheral arterial disease, coronary artery disease, and carotid stenosis than their counterparts selected for the transfemoral approach.^27^ More research is required to establish if the transapical approach is a modifiable risk factor for the development of delirium, and also if new cerebral emboli are relevant to the underlying mechanism of delirium TAVI patients. Although the evidence is not specific to TAVI, an association between microemboli, new strokes and postoperative neurocognitive decline has not been identified in patients who underwent cardiovascular interventions.^28^ An emerging theory attributes postoperative delirium and cognitive decline to underlying cerebrovascular disease in cardiovascular surgery patients.^28^

A potentially modifiable risk factor for the development of delirium in patients who underwent TAVI is acute kidney injury (over 5 times the risk, as compared with no kidney injury). While there is no standard protocol for the prevention of acute kidney injury during TAVI, a recent review has identified that adequate hydration and the avoidance of nephrotoxic medications remain the mainstay of preventative therapy.^29^ However, overhydration must also be avoided as it may lead to congestive heart failure.^29^ Therefore, further research is required.

Carotid artery disease was the only preprocedural risk factor for delirium following TAVI to have a large effect size (4 times the risk, as compared with no carotid artery disease) in the present study. Pooled figures also identified that a history of hypertension was significantly associated with the development of delirium in patients who underwent TAVI procedure. Both carotid artery disease and hypertension are known risk factors for delirium in patients who underwent cardiac surgery.^30^ TAVI patients with a history of prior cognitive impairment were also at increased risk of developing of delirium following their procedure (2 times the risk). Prior cognitive impairment is an established risk factor for the development of delirium across several hospital settings.^19^

The homogeneity of the age of TAVI patients may help to explain why increased age was not found to be a preprocedural risk factor for the development of delirium within this population. While male sex, American Society of Anesthesiologists classification > 3 and New York Heart Association Class of Heart Failure III-IV were not found to be risk factors for the development of delirium in this review of TAVI patients, they have been found to be risk factors for delirium in other hospital settings.^19^

The limitations of this systematic review are firstly that the review only included studies published in English. Secondly, due to the scarcity of literature, the review included all 9 studies reporting on the prevalence of delirium, despite no studies being adequately powered to detect prevalence of delirium in TAVI patients. Overall, the methodological quality of the studies assessed using the JBI Critical Appraisal Checklist for Studies Reporting Prevalence Data^9^ was relatively mediocre. The mean score was 5 of 9. However it is acknowledged that the prevalence of delirium was not the primary focus of the 9 cohort studies.

**Figure 3:**
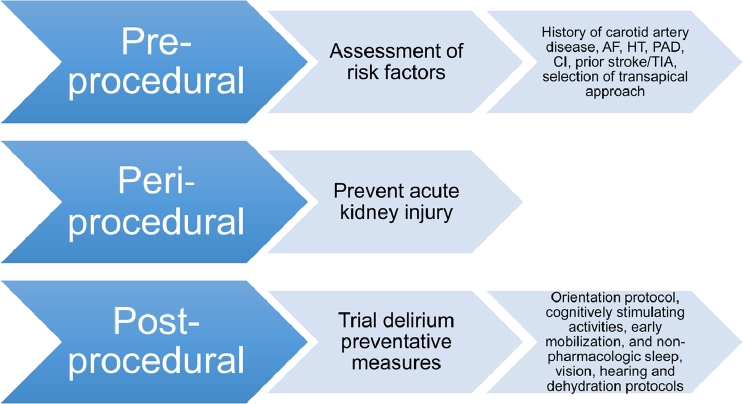
Clinical application of risk factors for postprocedural delirium in TAVI patients to consider. AF = atrial fibrillation; CI = cognitive impairment; HT = hypertension; PAD = peripheral artery disease; TIA = transient ischemic attack.

This systematic review has implications for clinical practice for patients who underwent TAVI and have been summarized in Figure 3. Proven delirium preventative measures,^6^ should be considered as part of postprocedural care for patients who undergo transapical approach TAVI, with a history of carotid artery disease, cognitive impairment, atrial fibrillation, stroke/TIA, peripheral artery disease, and hypertension. Where possible, acute kidney injury should be prevented in patients who underwent TAVI, as this is a modifiable risk factor for the development of delirium.^19^

In terms of implications for future research, given the pooled prevalence of delirium following TAVI is 23%, further studies examining the prevalence of delirium following TAVI procedure should have a sample size of at least 385 participants to be adequately powered (see Supplement Table 1 for calculations). Primary studies should ensure delirium is assessed daily for a minimum of 2 days following TAVI as this is the most common time to develop delirium.^16^, ^17^ Future research on risk factors for delirium following TAVI procedure should include modifiable risk factors that have been identified in other hospital populations including dehydration, electrolyte abnormalities, liver failure, urinary tract infection, pneumonia, physical restraints, bladder catheters, polypharmacy, hearing and vision.^19^ In addition, the effectiveness of delirium preventative measures in elderly hospitalized adults^6^ could be evaluated in the TAVI population.

## Disclosures

The authors have no relevant disclosures.

## Appendix Supplementary materials

Image, application 1
