# Supplementary material for: Meta-analysis of Prevalence and Risk Factors for Delirium After Transcatheter Aortic Valve Implantation
Source: Am J Cardiol. 2018 Dec 1;122(11):1917–23. doi: 10.1016/j.amjcard.2018.08.037 (PMC6269593; doi:10.1016/j.amjcard.2018.08.037)
Supplement: Supplementary file 1 [file mmc1.doc]

**Supplemental materials**

**Supplemental Methods**

**Search Terms by database:**

**Pubmed:**

(TAVR OR TAVI OR "transcatheter aortic valve" OR transcatheter aortic valve replacement[mh] OR "aortic valve replacement") AND (cogn* OR delirium OR delirium[mh] OR dementia OR dementia[mh] OR cognition disorders[mh] OR cognition[mh])

**CINAHL:**

(MM "Heart Valve Prosthesis Utilization" OR TI TAVR OR AB TAVR OR TI TAVI OR AB TAVI OR TI “transcather aortic valve” OR AB “transcatheter aortic valve” OR TI “aortic valve replacement” or AB “aortic valve replacement”) AND (MM “cognition” OR MM “delirium” OR TI “delirium” OR AB “delirium” OR TI “cognition” OR AB “cognition” OR MM “dementia” OR TI “dementia” OR AB “dementia”)

**Cochrane library:**

(TAVR OR TAVI OR “transcatheter aortic valve” OR “aortic valve replacement”) AND (cogn* OR delirium OR dementia)

**PsycInfo:**

**(**TAVR or TAVI or “transcatheter aortic valve” or “aortic valve replacement”).mp. and (cogn*.mp. or exp DELIRIUM/ or delirium.mp. or exp DEMENTIA/ or dementia.mp.)

**Embase:**

(('transcatheter aortic valve implantation'/syn) OR ('TAVI') OR ('TAVR') OR ('aortic valve replacement')) AND ((‘cogn*’) OR (‘cognition’/syn) OR ('delirium'/syn) OR (‘delirium’) OR (‘dementia’/syn) OR (‘dementia’)) AND ([embase]/lim NOT [medline]/lim)

**Supplemental Tables:**

Table 1: Critical appraisal scores for case studies meeting eligibility for inclusion for the review using the Joanna Briggs Institute Critical Appraisal Checklist.^1^

|  | **Abawi et al. 2016^2^** | **Assmann et al. 2016^3^** | **Eide et al. 2016^4^** | **Erdoes et al. 2012^5^** | **Fanning et al. 2016^6^** | **Huded et al. 2016^7^** | **Maniar et al. 2016^8^** | **Sharma et al. 2016^9^** | **Tse et al. 2015^10^** |
| --- | --- | --- | --- | --- | --- | --- | --- | --- | --- |
| 1. Was the sample frame appropriate to address the target population? | *Yes* | *Yes* | *Yes* | *Yes* | *Yes* | *Yes* | *Yes* | *Yes* | *Yes* |
| 2. Were study participants sampled in an appropriate way? | *Yes* | *Yes* | *Yes* | *Yes* | *Yes* | *Yes* | *Yes* | *Yes* | *Yes* |
| 3. Was the sample size adequate?* | *No* | *No* | *No* | *No* | *No* | *No* | *No* | *No* | *No* |
| 4. Were the study subjects and the setting described in detail? | *Yes* | *Yes* | *Yes* | *Yes* | *Yes* | *Yes* | *Yes* | *Yes* | *Yes* |
| 5. Was the data analysis conducted with sufficient coverage of the identified sample? | *Yes* | *Unclear* | *Unclear* | *Unclear* | *Unclear* | *Yes* | *Yes* | *Unclear* | *Unclear* |
| 6. Were valid methods used for the identification of the condition? | *Yes* | *Yes* | *Yes* | *Yes* | *Yes* | *Yes* | *Yes* | *Yes* | *Yes* |
| 7. Was the condition measured in a standard, reliable way for all participants? | *Unclear* | *Unclear* | *Unclear* | *Unclear* | *Unclear* | *Unclear* | *Unclear* | *Unclear* | *Unclear* |
|  | **Abawi et al. 2016^2^** | **Assmann et al. 2016^3^** | **Eide et al. 2016^4^** | **Erdoes et al. 2012^5^** | **Fanning et al. 2016^6^** | **Huded et al. 2016^7^** | **Maniar et al. 2016^8^** | **Sharma et al. 2016^9^** | **Tse et al. 2015^10^** |
| 8. Was there appropriate statistical analysis? | *No* | *No* | *No* | *Not applicable* | *No* | *No* | *Yes* | *No* | *No* |
| 9. Was the response rate adequate, and if not, was the low response rate managed appropriately? | *Yes* | *Yes* | *Yes* | *Unclear* | *Yes* | *Yes* | *Yes* | *Yes* | *Yes* |
| **Total score:** | **6/9** | **5/9** | **5/9** | **4/8** | **5/9** | **6/9** | **7/9** | **5/9** | **5/9** |

***** The following formula was used to determine whether or not the included studies utilized an adequate sample size;
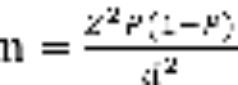
 where z is the z statistic for a level of confidence, P is the expected prevalence or proportion, and d is the precision. Where the estimated prevalence is unknown (as a meta-analysis on the prevalence of delirium following TAVI has not been conducted previously), conservative assumptions are preferable (up to 50%).^11^ Thus, assuming 5% precision with 95% CI, an adequately powered study would have a sample size of at least 385 participants.

Table 2: Forest plots and heterogeneity chi^2^  for pre- and peri-procedural variables for the development of delirium following TAVI.

| **Variable** | **Forest Plot** | **Heterogeneity Chi^2^** |
| --- | --- | --- |
| Age* | 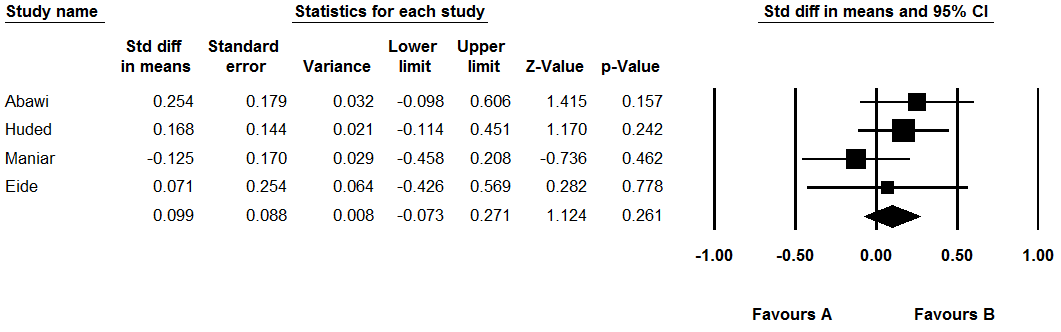 | 2.73, df=3, p=0.435. |
| ASA | 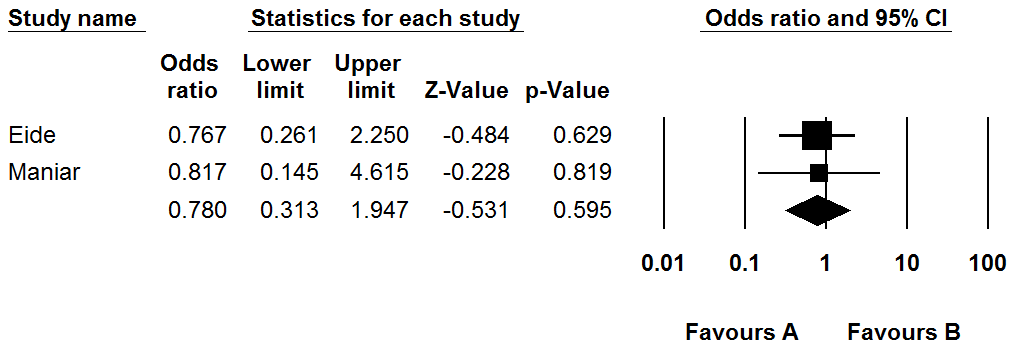 | 0.00, df=1,p=0.951 |
| AF | 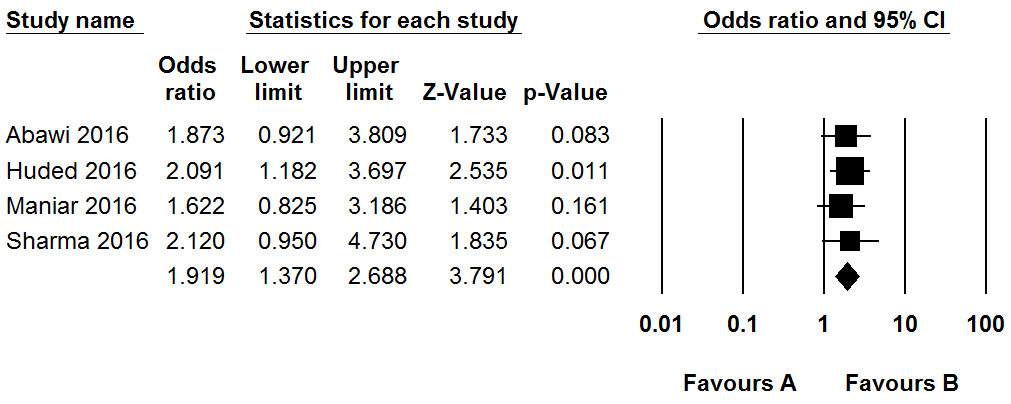 | 0.39, df=3, p=0.943 |
| AVA* | 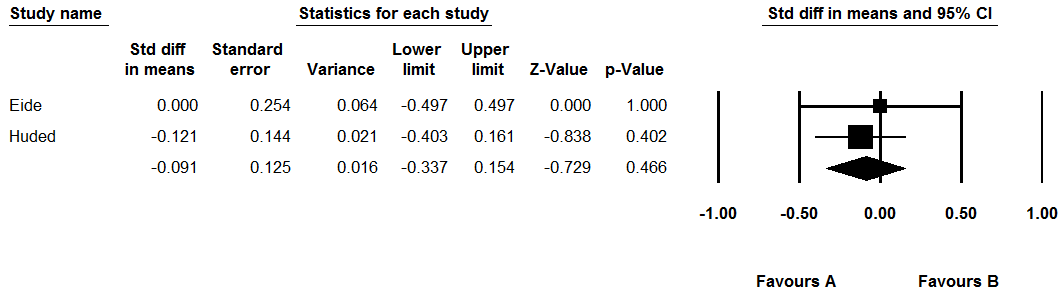 | 0.17, df=1, p=0.679 |
| BMI* | 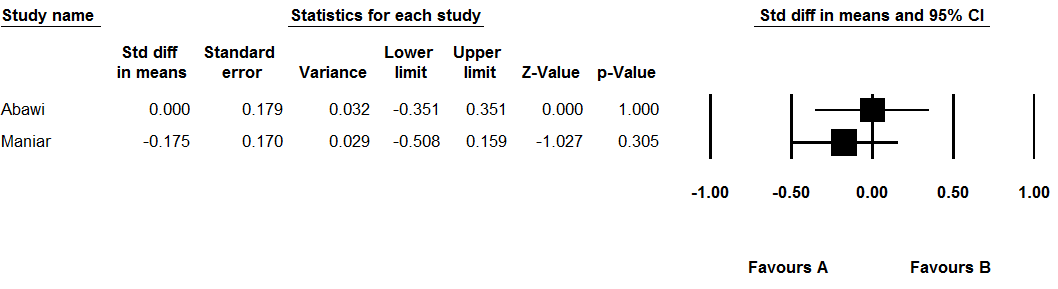 | 0.50, df1, p=0.480 |
| Carotid artery disease | 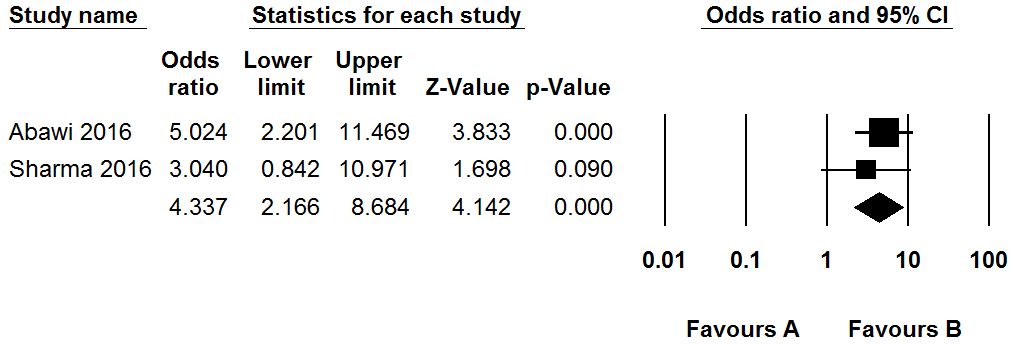 | 0.42, df=1, p=0.519 |
| CI | 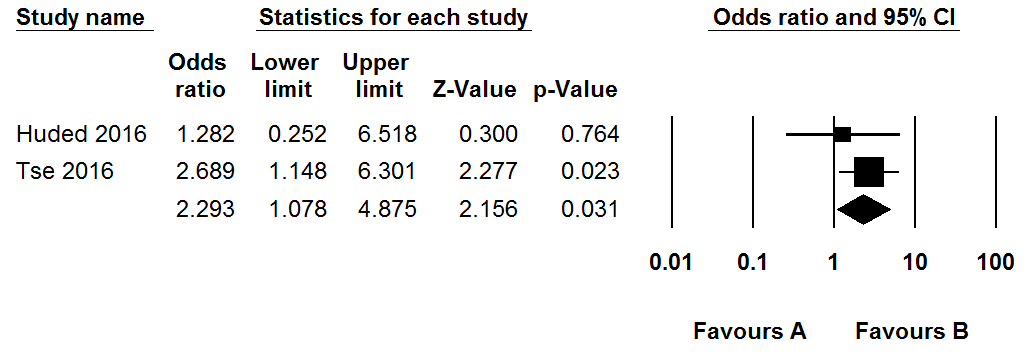 | 0.63, df=1, p=0.429 |
| COPD | 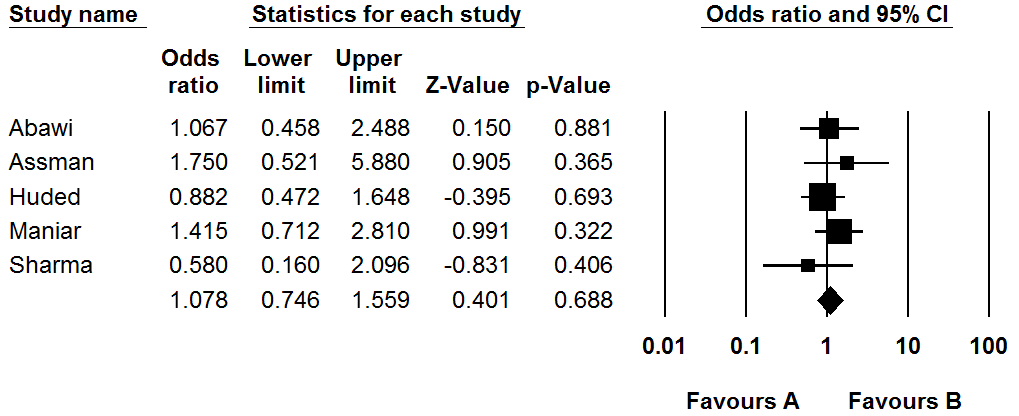 | 2.51, df=4, p=0.643 |
| CAD | 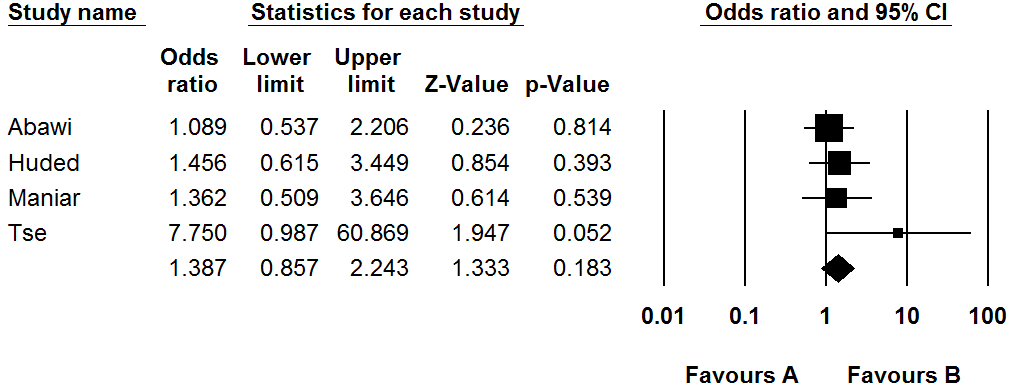 | 3.14, df=3, p=0.370 |
| Diabetes | 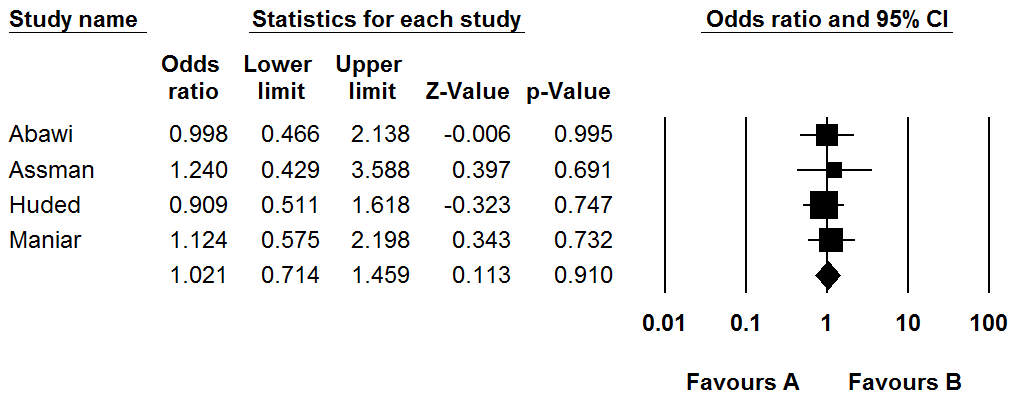 | 0.37, df=3, p=0.947 |
| EuroSCORE* | 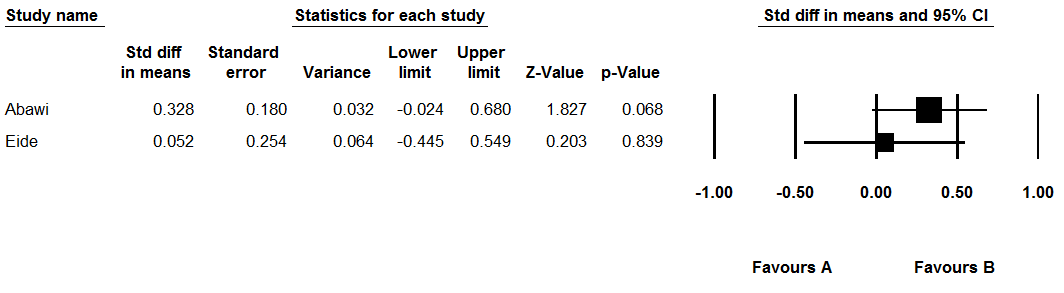 | 0.79, df=1, p=0.373 |
| GFR * | 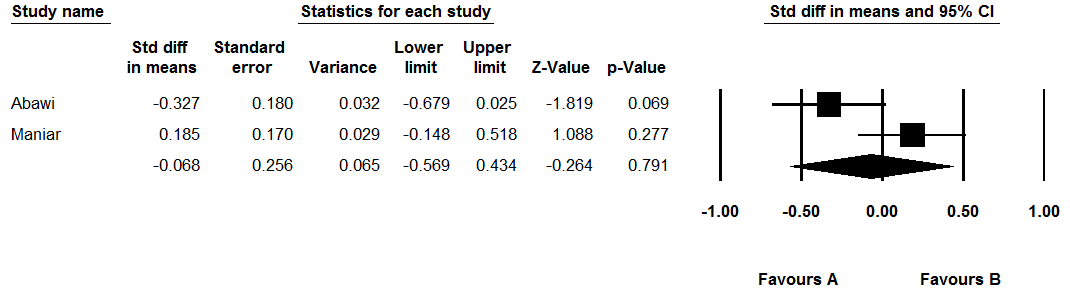 | 4.28, df=1, p=0.039 |
| Haemoglobin* | 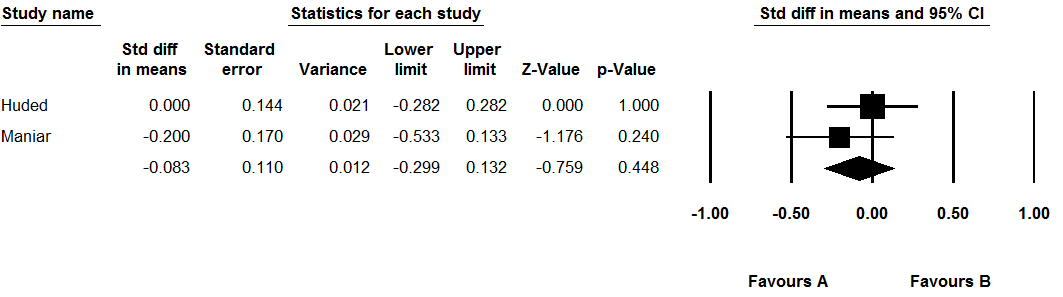 | 0.81, df=1, p=0.369 |
| Hypertension | 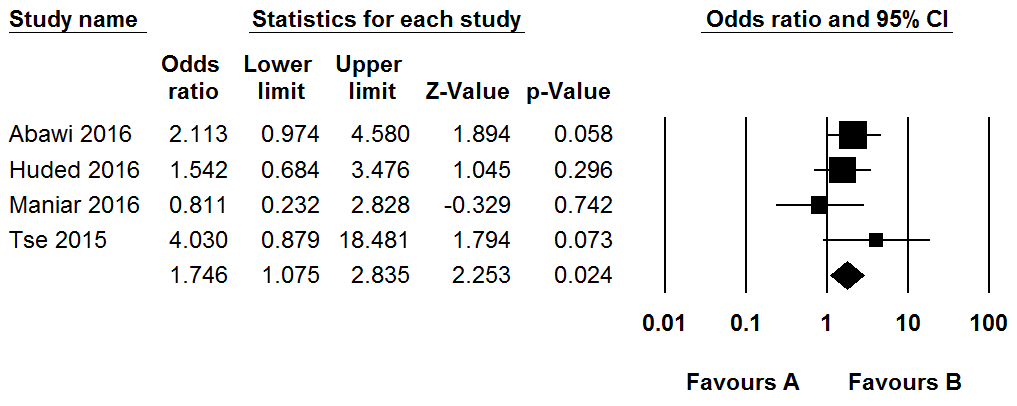 | **2.93, df=3, p=0.403** |
| LVEF* | 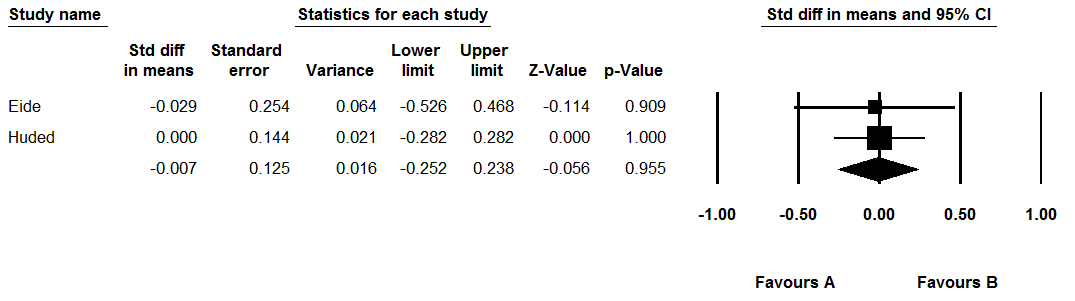 | 0.01, df=1, p=0.921 |
| Men | 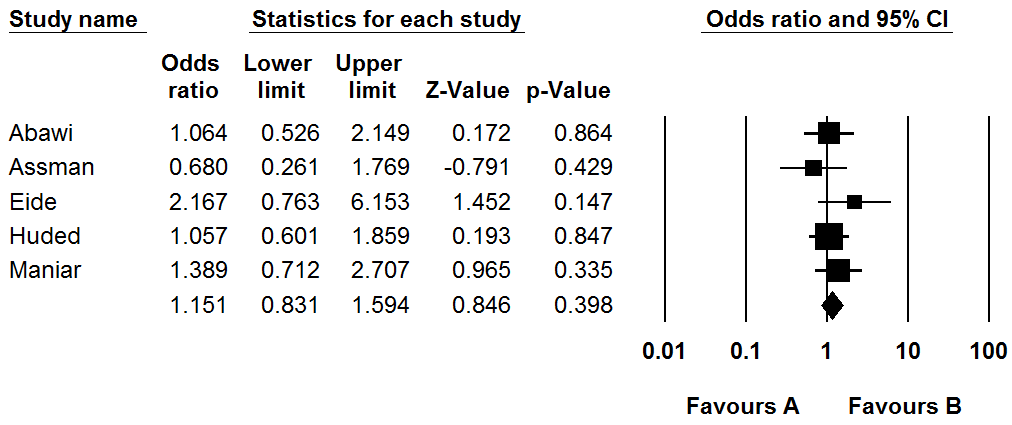 | 3.01, df=4, p=0.555 |
| NYHA III-IV | 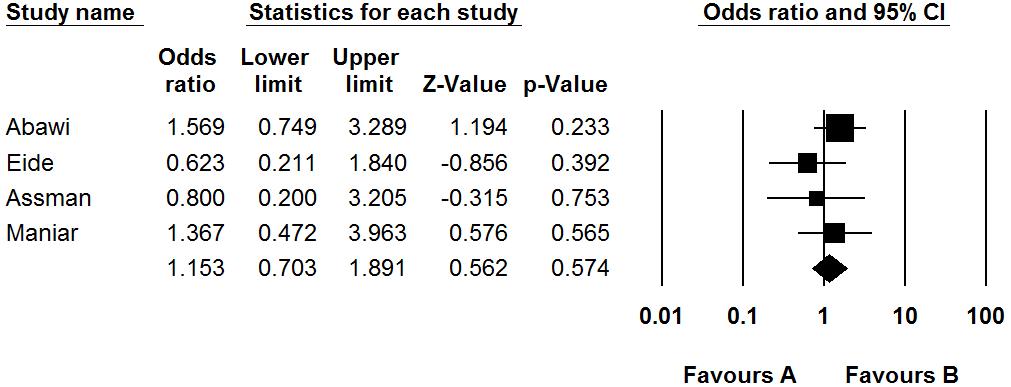 | 2.27, df=3, p=0.518 |
| Peripheral artery disease | 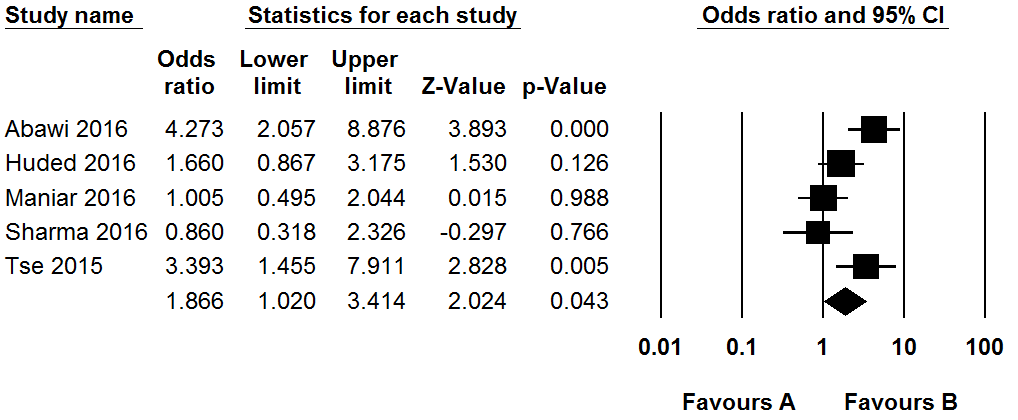 | **12.22, df=4, p=0.016** |
| Prior CABG | 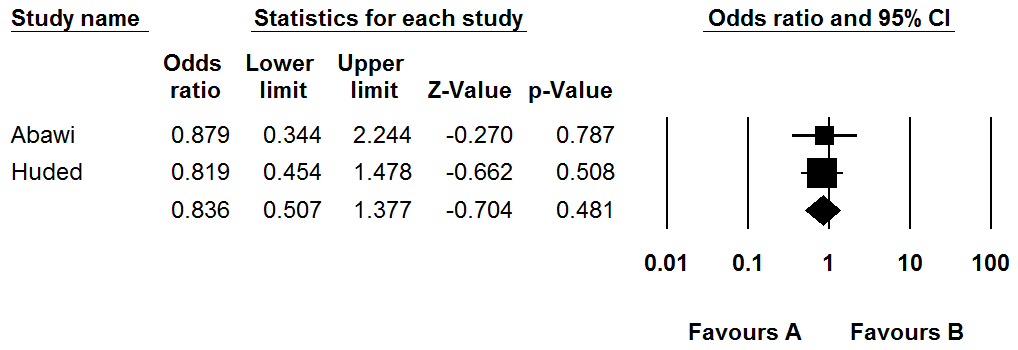 | 0.02, df=1, p=0.901 |
| Stroke/TIA | 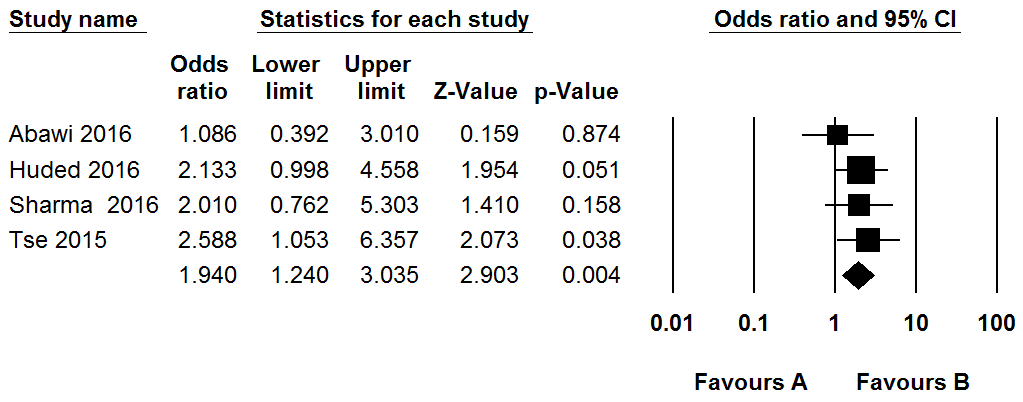 | **1.70, df=3, p=0.636** |
| Acute Kidney Injury | 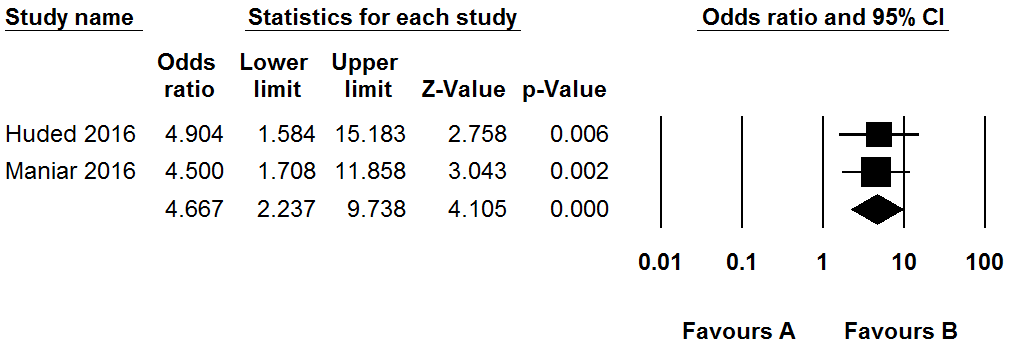 | **0.01, df=1, p=0.910** |
| Transapical approach | 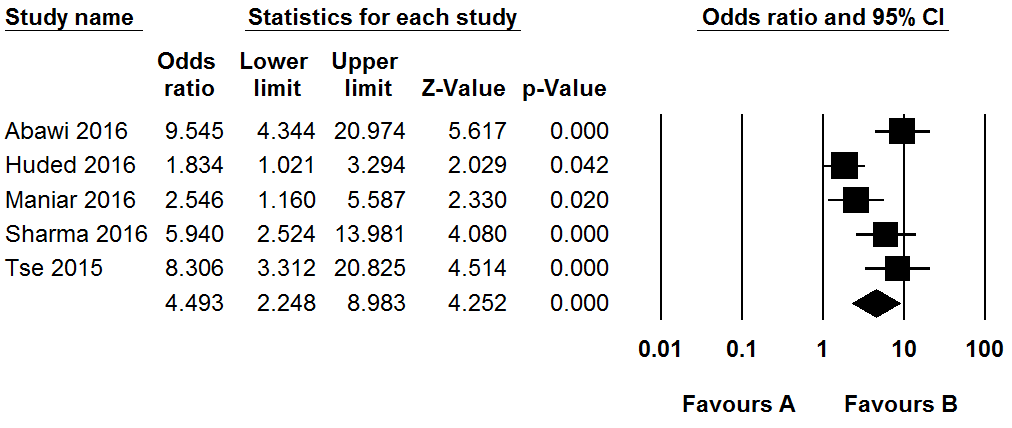 | **16, df=4, p=0.003** |

ASA=American Society of Anaesthesiologists Physical Status Class 4, AF= atrial fibrillation, AVA= aortic valve area, BMI= Body Mass Index, CABG= coronary artery bypass grafting, CAD=coronary artery disease, CI= Cognitive Impairment, COPD= chornic obstructive pulmonary disease, EuroSCORE=European system for cardiac operative risk evaluation, GFR=Glomerular filtration rate, LVEF= left ventricular ejection fraction, NYHA III-IV =New York Heart Association Class of Heart Failure III-IV, TAVI= transcatheter aortic valve implantation, and TIA= transient ischemic attack.

**Supplemental References**

1. Munn Z, Moola S, Riitano D, Lisy K. The development of a critical appraisal tool for use in systematic reviews addressing questions of prevalence. *IJHPM* 2014;3:123-128.
2. Abawi M, Nijhoff F, Agostoni P, Emmelot-Vonk MH, De Vries R, Doevendans PA, Stella PR. Incidence, Predictive Factors, and Effect of Delirium after Transcatheter Aortic Valve Replacement. *JACC Cardiovasc Interv* 2016;9:160-168.
3. Assmann P, Kievit P, van der Wulp K, Verkroost M, Noyez L, Bor H, Schoon Y. Frailty is associated with delirium and mortality after transcatheter aortic valve implantation. *Open heart* 2016;3:1-8.
4. Eide LS, Ranhoff AH, Fridlund B, Haaverstad R, Hufthammer KO, Kuiper KK, Nordrehaug JE, Norekval TM. Delirium as a Predictor of Physical and Cognitive Function in Individuals Aged 80 and Older After Transcatheter Aortic Valve Implantation or Surgical Aortic Valve Replacement. *J Am Geriatr Soc* 2016; 64:1178-186.
5. Erdoes G, Basciani R, Huber C, Stortecky S, Wenaweser P, Windecker S, Carrel T, Eberle B. Transcranial Doppler-detected cerebral embolic load during transcatheter aortic valve implantation. *Eur J Cardiothorac Surg* 2012;41:778-183.
6. Fanning JP, Wesley AJ, Walters DL, Eeles EM, Barnett AG, Platts DG, Clarke AJ, Wong AA, Strugnell WE, O'Sullivan C, Tronstad O, Fraser JF. Neurological Injury in Intermediate-Risk Transcatheter Aortic Valve Implantation. *J Am Heart Assoc* 2016; 5:1-8.
7. Huded CP, Huded JM, Sweis RN, Ricciardi MJ, Malaisrie SC, Davidson CJ, Flaherty JD. The impact of delirium on healthcare utilization and survival after transcatheter aortic valve replacement. *Catheter Cardiovasc Interv* 2016;00:00-00.
8. Maniar H, Lindman B, Escallier K, Avidan M, Novak E, Melby S, Damiano M, Lasala J, Quader N, Rao R, Lawton J, Moon M, Helsten D, Pasque M, Damiano R, Zajarias A. Delirium after surgical and transcatheter aortic valve replacement is associated with increased mortality. *J Thorac Cardiovasc Surg* 2016; 151:815-823.
9. Sharma V, Katznelson R, Horlick E, Osten M, Styra R, Cusimano RJ, Carroll J, Djaiani G. Delirium after transcatheter aortic valve implantation via the femoral or apical route. *Anaesthesia* 2016;71:901-907.
10. Tse L, Bowering JB, Schwarz SK, Moore RL, Burns KD, Barr AM. Postoperative delirium following transcatheter aortic valve implantation: a historical cohort study. *Can J Anaesth* 2015;62:22-30.
11. Macfarlane SB. Conducting a descriptive survey: 2. Choosing a sampling strategy. *Trop Doct* 1997;27:14-21.
